# Raw milk and fecal microbiota of commercial Alpine dairy cows varies with herd, fat content and diet

**DOI:** 10.1371/journal.pone.0237262

**Published:** 2020-08-06

**Authors:** Francesca Albonico, Claudia Barelli, Davide Albanese, Mattia Manica, Erika Partel, Fausta Rosso, Silvia Ripellino, Massimo Pindo, Claudio Donati, Alfonso Zecconi, Michele Mortarino, Heidi C. Hauffe

**Affiliations:** 1 Department of Biodiversity and Molecular Ecology, Research and Innovation Centre, Fondazione Edmund Mach, San Michele all’Adige, Trento, Italy; 2 Department of Veterinary Medicine, Universiy of Milan, Milan, Italy; 3 Department of Biology, University of Florence, Sesto Fiorentino, Firenze, Italy; 4 Unit of Computational Biology, Research and Innovation Centre, Fondazione Edmund Mach, San Michele all’Adige, Trento, Italy; 5 Technology Transfer Centre, Fondazione Edmund Mach, San Michele all’Adige, Trento, Italy; 6 Department of Genomics and Biology of Fruit Crops, Research and Innovation Centre, Fondazione Edmund Mach, S. Michele all’ Adige (TN), Trento, Italy; 7 Department of Biomedical, Surgical and Dental Sciences, Università degli Studi di Milano, Milan, Italy; The University of Sydney, AUSTRALIA

## Abstract

The factors that influence the diversity and composition of raw milk and fecal microbiota in healthy commercial dairy herds are not fully understood, partially because the majority of metataxonomic studies involve experimental farms and/or single factors. We analyzed the raw milk and fecal microbiota of 100 healthy cows from 10 commercial alpine farms from the Province of Trento, Italy, using metataxonomics and applied statistical modelling to investigate which extrinsic and intrinsic parameters (e.g. herd, diet and milk characteristics) correlated with microbiota richness and composition in these relatively small traditional farms. We confirmed that Firmicutes, *Ruminococcaceae* and *Lachnospiraceae* families dominated the fecal and milk samples of these dairy cows, but in addition, we found an association between the number of observed OTUs and Shannon entropy on each farm that indicates higher microbiota richness is associated with increased microbiota stability. Modelling showed that herd was the most significant factor affecting the variation in both milk and fecal microbiota composition. Furthermore, the most important predictors explaining the variation of microbiota richness were milk characteristics (i.e. percentage fat) and diet for milk and fecal samples, respectively. We discuss how high intra-herd variation could affect the development of treatments based on microbiota manipulation.

## Introduction

For at least the last 5 000 years, many human populations have supplemented their diet by drinking ruminant milk [1]. It has been known for some time that raw milk from domesticated animals has its own unique endogenous microbial community, which impacts the health of both human and livestock offspring [2, 3]. It has also been shown that during bacterial infections of the mammary glands, the alpha and beta diversities (richness and composition, respectively) of raw milk microbiota may be drastically reduced but remains resilient, able to restore itself and offer protection against invading pathogens, in some cases even without antimicrobial treatment [4, 5]. Given their role in animal health and productivity, as well as food quality and safety, characterization of raw milk and gastrointestinal microbiota has become a focus of interest in livestock research [2, 3, 6]. Thanks to the advent of pyrosequencing, in-depth characterization of microbial communities, both culturable and unculturable components, is now possible. Although molecular approaches can also introduce their own forms of bias, such as the ability to detect both viable and inviable bacteria, they currently provide the most potent tools available for determining the richness and composition of human and animal microbiomes [3, 7, 8].

Despite widespread use of this relatively new technology, very little is still documented about the richness, composition and variation of ruminant raw milk microbiota in healthy dairy livestock in commercial (as opposed to experimental) farm environments. In one of the few studies of commercial dairy herds, it has been shown that alpine and lowland herds had distinct microbiota from each other [9]. In another study, a large intra-individual variation was detected between samples of healthy cows from two different herds [10]. Even less is known about the factors that influence variation in microbiota, and the impact of this variation on milk characteristics and therefore, on milk quality [3]. In dairy cows from experimental herds, high concentrate diet may lead to changes in milk microbiota composition (but not richness), with a greater abundance of pathogenic and psychotropic bacteria associated with mastitis and poor food quality, respectively; however, this study included only four individuals [11]. Moreover, using pooled samples from one herd of 60 experimental dairy cows, housing (indoor vs outdoor) and teat preparation have demonstrated to influence the richness and composition of milk microbiota [12]. However, factors affecting the variation of milk microbiota in individual healthy cows from commercial herds has not been explored.

Because fecal collection, unlike ruminal samples, is non-invasive, practical, and widely used for sampling animals repeatedly, fecal microbiota is commonly used as a proxy for gastrointestinal microbiota in livestock studies [13–18]. The fecal microbiota of cattle not only reflects condition and productivity, but a better understanding of its richness and composition is important for understanding how the transmission of foodborne pathogens could be decreased or avoided [18, 19]. While fecal microbiota in cattle has been already described [20], factors that influence their richness and composition have only been investigated in a handful of studies. Two studies in experimental animals [21, 22] concluded that, unlike the ruminal microbiota, fecal microbiota was not influenced by diet (although the opposite was found for beef cattle [13, 19]). In the few studies of commercial dairy herds, Tang and colleagues [23] found that feeding management and country of origin was more important than diet in 18 silage-fed dairy cows (six herds), while Xu and colleagues [24] also noted that fecal microbiota composition changed with probiotic treatment. In addition, individual variation in fecal microbiota, even in the same farm, has been noted [20, 25]. Differences observed in studies that analyzed similar farms from the same regions using similar methodologies suggest that there may be additional influences on this microbiota [23, 26].

Since raw milk and gut microbiota are not independent, but interact with each other, milk yield and quality may be influenced by the microbial composition of the rumen and feces and *vice versa* [24, 27–29]. For example, raw milk microbiota may originate from the entero-mammary pathway [30, 31] as well as from exogenous sources through the teat apex, including the animal’s own skin, fecal matter, and the farm environment [2, 32]. On the other hand, the nutritional components and biological actions of milk include the establishment of the gut microbiota, as well as immunological and endocrine competence, which are important for the development of all mammalian offspring [3, 33–35]. However, up to now, factors potentially influencing raw milk and fecal microbiota have been considered singularly, and comparison of milk and fecal microbiota in the same healthy individuals from commercial herds has not yet been investigated. Therefore, by using metataxonomics, we investigated here the microbial community composition in raw milk and feces of typical alpine herds of dairy cows on commercial Trentino farms. We then explored which individual milk and environmental factors could be associated with microbiota variation. This is the first time, to our knowledge, that such factors have been considered together, using empirical data from commercial rather than experimental herds.

## Materials and methods

### Sample collection

Ten herds of typical mixed-breed dairy cows (i.e. Holstein Friesian, Brown Swiss, Pezzata Rossa Italiana) from farms in the Province of Trento (Italy) were selected for sampling. For five herds, cows were fed a traditional diet (TF) of dry forage, principally sun-dried locally cut meadow grass (abbreviated hereafter as: TF1, TF2, TF3, TF4, TF5), while five herds were provided with total mixed ration with silage, or unifeed (UF; hereafter as: UF1, UF2, UF3, UF4, UF5), with an overall forage to concentrate ratio of about 0.55:0.45. Other than this difference in diet, all the dairy herds were family-owned with a similar non-intensive, free-ranging management (<100 milking head per herd) with individual cubicles. The cows were milked twice a day by milking machine. During a period between April-July 2015, from each of the ten herds, ten primiparous cows at 90 to 180 days of lactation were chosen for sampling. Based on medical history and veterinary checks by EP (a registered veterinarian), all dairy cows were considered healthy and hence included in the current investigation. Animals that presented clinical signs suggestive of any disease were excluded from the study (S3 Table and S4 Table). No antibiotic treatments were administered 20–30 days to or during the sampling period.

### Raw milk and fecal sample collection

Using disposable gloves to handle the udder, immediately before each manual milk sampling, all four teats were cleaned and disinfected with cotton wool soaked in 100% ethanol. The initial three streams of milk from each teat were discarded, and two aliquots (pools of all four quarters) were collected from each cow. For microbiota analysis, a first aliquot (10 ml) was collected in a sterile 15 ml plastic tube (Starstedt, Verona, ITA). For standard milk analyses, a second aliquot (15 ml) was collected in the same type of tube with the addition of methylene blue. All milk samples were immediately refrigerated at 4°C and transported on ice to the Fondazione E. Mach within 3 hours of collection. The aliquots destined for metataxonomic analysis were immediately stored at -80°C until DNA extraction. The aliquots destined for standard analyses were kept refrigerated at 4°C and sent to the Associazione Regionale Allevatori della Lombardia (ARAL) laboratory (Crema, Italy), for a somatic cell count (SCC), total bacterial count (TBC) and percent fat, protein and lactose estimates. Twenty-four milk samples with an SCC (cells/ml milk) greater than 200 000 cells/ml (an indication of mastitis: [36]) were excluded from further analysis.

Fecal samples were collected non-invasively directly from the rectum of each cow using a disposable glove, immediately refrigerated at 4°C, and transported to the Fondazione E. Mach on ice, where they were aliquoted; at least one aliquot of 25 mg was stored at -80°C until DNA extraction, while another (7 g) was kept at 4°C until parasitological analysis.

### DNA isolation and purification

One milk sample from each cow was thawed on ice and homogenized by inverting the tubes three times. Total genomic DNA was isolated from a 1 ml aliquot using a commercially available kit (PowerMax Soil DNA Isolation Kit, MOBIO Laboratories Inc., Carlsbad, CA, USA) following manufacturer’s instructions with the following modifications: elution was carried out with 2 ml of buffer and following a 5-minute incubation of the columns with buffer at room temperature. One ml of eluted DNA preparation was further purified using the Amicon® Ultra-2 mL 30K centrifugal filters (Millipore, Cork, IRL). After a centrifugation at 7 500 g for 20 min, concentrated DNA was obtained in a final volume of 50 μL. Concentration of isolated DNA was evaluated using the Qubit dsDNA High Sensitivity (HS) Assay Kit (Invitrogen, ThermoFisher Scientific, Waltham, MA, USA). Total DNA was extracted from one 25 mg fecal sample per cow using a Mixer Mill MM 200 (Retsch, Haan, Germany) and the PowerSoil DNA Isolation Kit (MO BIO Laboratories Inc., Carlsbad, CA, USA) following manufacturer's instructions. Concentration of isolated DNA was evaluated as above.

### Library construction and sequencing

The V3-V4 hypervariable regions of the bacterial 16S rRNA gene were amplified using specific primers 341F (5’ TCGTCGGCAGCGTCAGATGTGTATAAGAGACAG) and 805R (5’ GTCTCGTGGGCTCGGAGATGTGTATAAGAGACAG) with overhang Illumina adapters. The PCR reactions for each sample were carried out in a total volume of 25 μl containing 0.2 μM of each primer, 12.5 μl of Kapa HiFi Ready Mix (Kapa Biosystem, Wilmington, MA, USA) and 12.5 ng of genomic DNA, as suggested by the Illumina protocols [37]. Thermal cycling was performed on a GeneAmp™ PCR System 9700 instrument (Thermo Fisher Scientific, MA, USA) with two different thermal protocols depending on sample type. For milk samples: initial denaturation at 95°C for 5 min, followed by 35 cycles at 95°C for 30 s, 57°C for 30 s and a final extension at 72°C for 5 min. For fecal samples: initial denaturation at 95°C for 5 min, followed by 25 cycles at 95°C for 30 s, 55°C for 30 s, extension at 72°C for 30 s, and a final extension at 72°C for 5 min. At least one negative control was included for each extraction and each PCR run. In addition, genomic DNA from the Microbial Mock Community B (Staggered, Low Concentration) v5.2L (BEI Resources, Manassas, VA, USA) was amplified once for each sample type to act as a positive control during sequencing. The PCR products were checked on 1.5% agarose gel and free primers and primer dimers were removed using the Agencourt AMPure XP system (Beckman Coulter, Brea, CA, USA) following the manufacturer’s instructions. Subsequently, dual indices and Illumina sequencing adapters Nextera XT Index Primer (Illumina) were attached using seven PCR cycles [37]. After purification with the Agencourt AMPure XP system (Beckman), the final libraries were analyzed on a Typestation 2200 platform (Agilent Technologies, Santa Clara, CA, USA) and quantified using the Quant-IT PicoGreen dsDNA assay kit (Thermo Fisher Scientific) on the Synergy2 microplate reader (Biotek, Winooski, VT, USA). Finally, all libraries were pooled in an equimolar way in a final amplicon library and analyzed on a Typestation 2200 platform (Agilent Technologies). The barcoded library was sequenced on an Illumina® MiSeq (PE300) platform (MiSeq Control Software 2.5.0.5 and Real-Time Analysis software 1.18.54.0).

### Bioinformatic processing

16S rRNA gene sequences were processed using the open-source MICCA (v1.6) software [38]. Overlapping paired-end reads were assembled using the procedure described by Edgar and Flyvbjerg in 2015 [39]. Pairs with an overlap length smaller than 80 bp and with more than 32 mismatches were discarded. After primer trimming, forward reads shorter than 400 bp and with an expected error rate higher than 0.75% were discarded. Reads with less than 60% identity to the sequences present in the Greengenes database (v. 13_8) [40] clustered at 85% similarity were discarded. OTUs were inferred using a *de novo*, greedy strategy using a cutoff of 97% similarity. Resulting representatives of each OTU were classified using the Ribosomal Database Project classifier (RDP classifier, version 2.12 [41]).

### Parasitological analysis of feces

For each fresh fecal sample, the presence of helminth eggs and protozoan cysts was assessed using a standard floatation procedure. Each sample was mixed in a pestle with 30 ml floating medium (a saturated solution of sugar and sodium nitrate), passed through a 1 mm filter and centrifuged at 4 000 rpm for five minutes in a 15 ml glass tube. Two tubes were prepared for each sample. After centrifugation, floating medium was added until the tubes were filled (approx. 1 ml). Eggs were collected from the surface of each tube using a glass slide. The slides were observed at 10X and 40X magnification using an optical dissecting microscope, and eggs were identified to species using keys provided by Soulsby [42] and Sloss and Kemp [43].

### Data processing and statistical analyses

Downstream analyses were performed using R with the *phyloseq* [44] and *vegan* packages. After subclinical mastitis milk samples were removed (see above), the remaining samples were rarefied (without replacement) at 2 000 reads per sample resulting in seven additional samples being discarded. Fecal samples were rarefied (without replacement) at 18 500 reads per sample resulting in one sample being discarded. Estimation of standard alpha and beta diversity indices, and Principal Coordinate Analysis (PCoA) were performed using the R *phyloseq* library.

A comparison of the variation in the Inverse Simpson diversity index values between milk and fecal samples was carried out using a Linear Mixed Model (LMM) fitted using the R statistical software version 3.5.0 [45] and the *lme4* package [46]. The response variable was the Inverse Simpson diversity index, the covariate was ‘sample type’, and ‘herd’ was included as random effect to take into account unknown differences in management that may occur between sampled herds. Secondly, two separate LMMs were developed to investigate which intrinsic and extrinsic parameters influence milk and fecal microbiota composition. For both these models, the response variable was the Inverse Simpson diversity index; ‘herd’ was included in the models as a random effect. For milk samples, the explanatory variables were initially i) diet (TF *vs*. UF); ii) calving date; iii) breed; iv) SCC; v) TBC; vi) percentage fat; vii) percentage protein; and viii) percentage lactose (S3 Table). For fecal samples, explanatory variables included i) to iii) as above and iv) presence of coccidia (other parasites rather than coccidia were rare in our samples) (S4 Table). Collinearity among independent variables was assessed using variance inflation factors (VIF) with a cut-off value of 2. Given the VIF results, variable viii) ‘percentage lactose’ was excluded from further analysis of the milk samples while variable iii) ‘breed’ was excluded from both models. Following the exclusion of collinear variables, the two full models were considered for further model selection. All quantitative variables were standardized, and following a multi-model inference procedure, all possible sub-models were compared and ranked using the Akaike Information Criterion (AICc) with small sample bias adjustment [47]. Model averaged values and the relative importance of each explanatory variable were computed over the whole model set. Finally, a direct comparison of Inverse Simpson diversity index values among herds was carried out by including ‘herd’ as a covariate in two new linear models (LM) after removing ‘diet’ due to collinearity.

### Ethics statement

This study was conducted with samples from ten commercial dairy farms situated in Trentino with permission from participating farmers. For on-farm non-invasive research, no ethical committee oversight is required under Italian and Provincial laws. Authors confirm they did not disturb the animals in any way, and both raw milk and fecal sampling was conducted using non-invasive methods in accordance with approved guidelines of the farm veterinarians. All applicable institutional and/or national guidelines for the care and use of animals were followed.

## Results

### Taxonomic classification of raw milk and fecal microbiota

**Fig 1:**
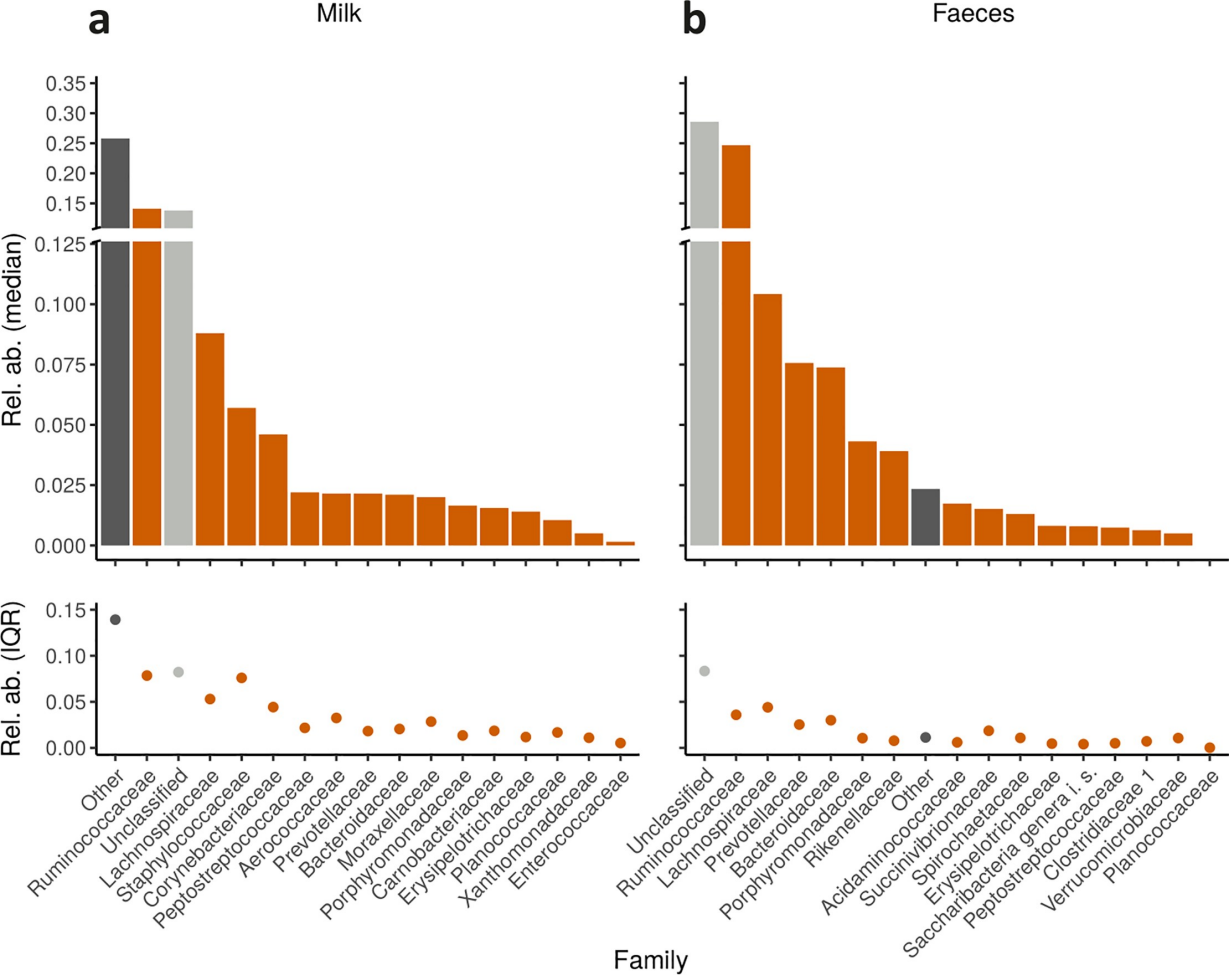
Taxonomic classification by family of raw milk and fecal microbiota from dairy cows. Relative abundances of the most abundant families of Alpine dairy cow raw milk (a) and fecal (b) samples. Median relative abundances are represented in the upper panels, while their variability expressed as interquartiles (IQR) are shown in the lower panels.

**Fig 2:**
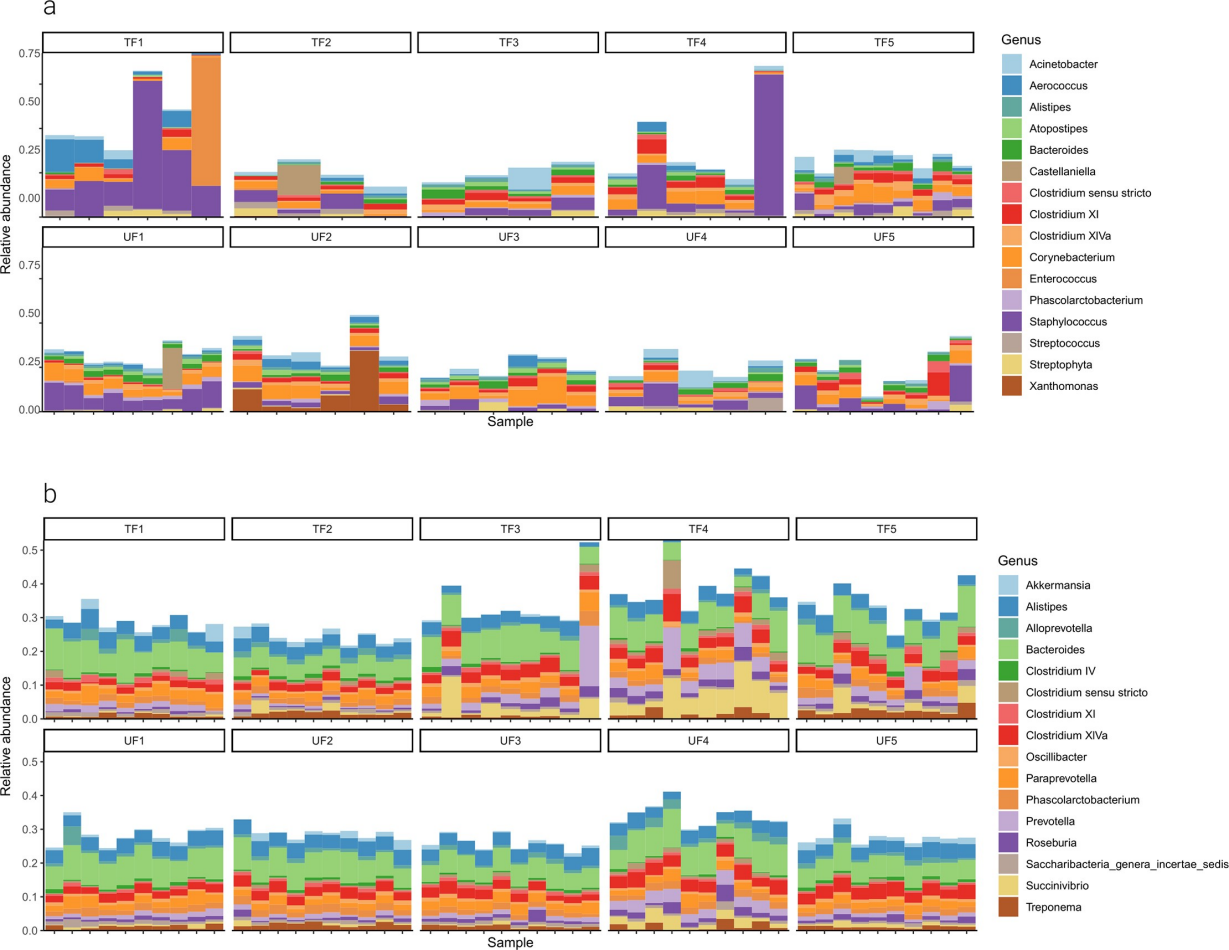
Taxonomic classification by genus of raw milk and fecal microbiota from dairy cows. Relative abundances of the most abundant genera of Alpine dairy cow raw milk (a) and fecal (b) samples across ten herds either fed traditionally (TF) or with unifeed (UF) within the Province of Trento, Italy.

Out of 76 milk samples, a total of 70 were successfully sequenced and after rarefaction, 63 were available for bioinformatic analyses. Among the 25 phyla taxonomically identified in raw milk samples, the dominant ones were Firmicutes (median abundance 55.3%), followed by Bacteroidetes (13.7%), Proteobacteria (11.6%) and Actinobacteria (11.3%). The remaining phyla were characterized by a relative abundance of less than 1% (S1 Table). At the family level (Fig 1A), *Ruminococcaceae* (median abundance 14.1%) were the most abundant, followed by *Lachnospiraceae* (8.8%) and *Staphylococcaceae* (5.7%). While dominant genera included *Staphylococcus* (median abundance 4.7%) and *Corynebacterium* (4.5%), followed by *Clostridium* XI (2.1%), *Bacteroides* (2.1%), *Clostridium* XIVa (1.8%), *Acinetobacter* (1.6%) (Fig 2A). Non-classified OTUs in milk at this level were 39.8%, far less than those in feces (65.2%).

All 100 fecal samples were successfully sequenced, and only one sample was removed after rarefaction. Sixteen phyla were taxonomically assigned in feces (S2 Table), with Firmicutes (median abundance 51.2%) and Bacteroidetes (38.4%) representing the two most dominant phyla, followed by Proteobacteria (2.6%) and Spirochaetes (1.3%). At this level, very few non-classified OTUs were detected (3.3% *vs*. 2.1% in raw milk). Similar to raw milk, *Ruminococcaceae* (24.7%) and *Lachnospiracee* (10.4%) were the two most abundant families in fecal samples (Fig 1B), followed by *Prevotellaceae* (7.6%) and *Bacteroidaceae* (7.4%). All other families were present at less than 5%; the non-classified OTUs at this level were 28.6% (*vs*. 13.8% in raw milk). The genera *Bacteroides* (7.3%), *Alistipes* (3.9%), *Clostridium* XIVa (2.7%), *Paraprevotella* (2.2%), *Phascolarctobacterium* (1.7%), *Prevotella* (1.5%), *Treponema* (1.3%) and *Roseburia* (1%) dominated (Fig 2B) fecal samples. The non-classified OTUs at the genus level were 65.2% (vs. 39.8% in raw milk).

### Microbiota richness of raw milk and fecal samples

**Fig 3:**
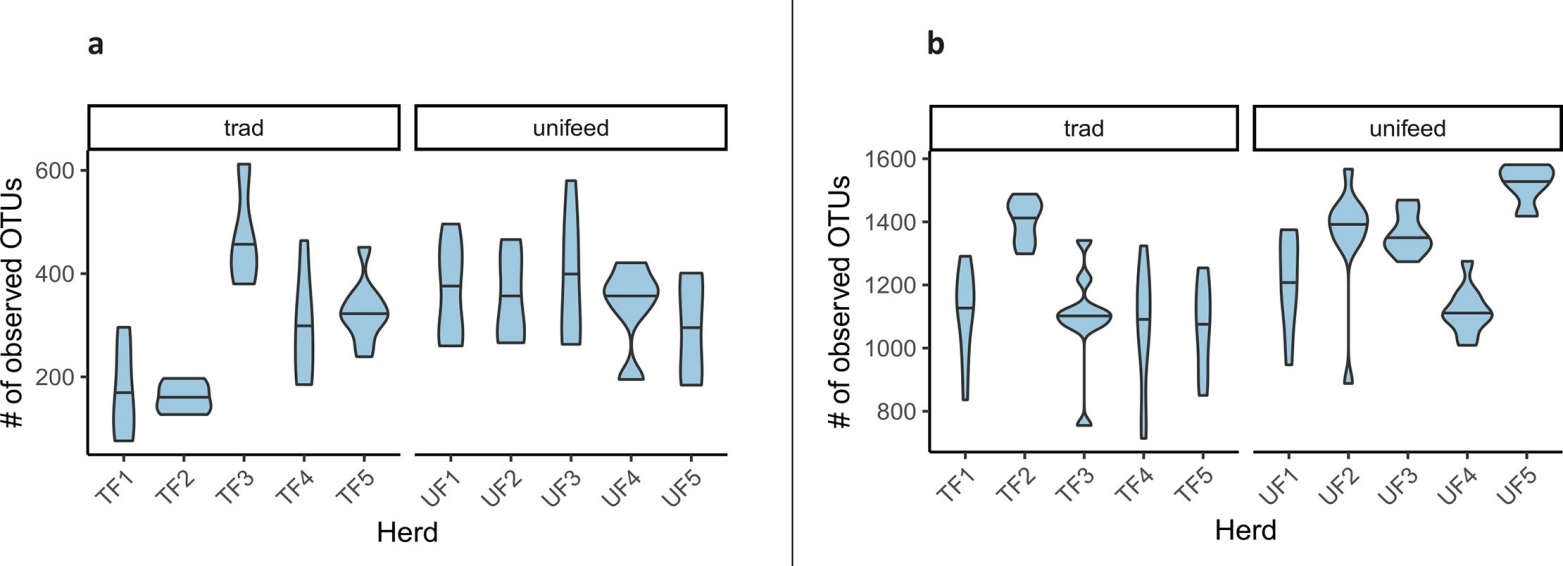
Estimates of microbiota richness. Violin plot of the total number of observed OTUs of Alpine dairy cow raw milk (a) and fecal (b) microbiota.

**Fig 4:**
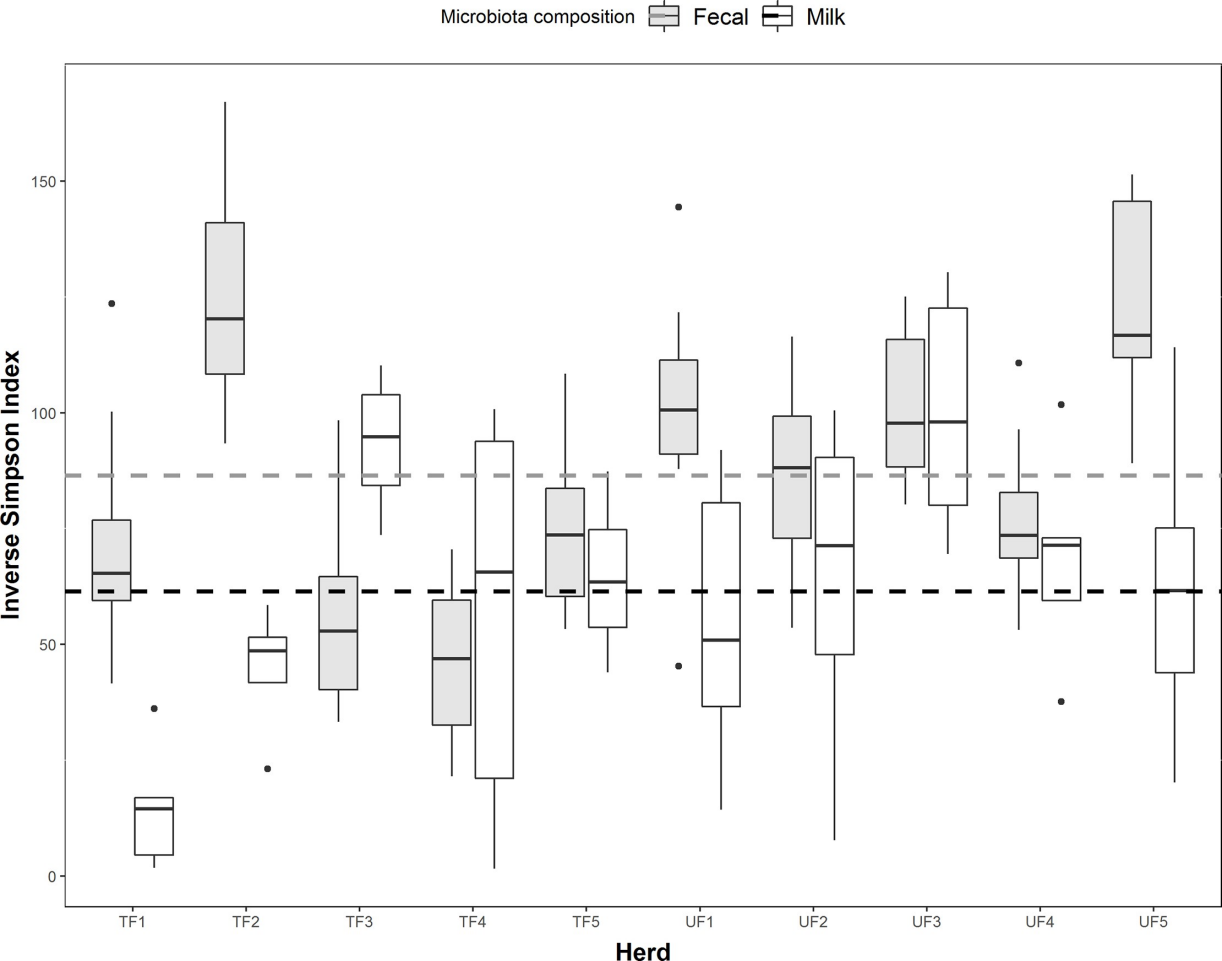
Estimates of microbiota richness of raw milk (white bars) and fecal samples (grey bars) of commercial Alpine dairy cows. Boxplot of observed values of the Inverse Simpson Index for milk and fecal microbiota across ten herds either fed traditionally (TF) or with unifeed (UF) in the Province of Trento, Italy. Horizontal dashed lines represent the mean values for each corresponding microbiota. Dots are observations not included between the whiskers.

Estimates of richness (alpha diversity) varied widely between individuals; the total number of observed OTUs in raw milk samples ranged from 76 to 609 (Fig 3A; S3 Table), while values of the Inverse Simpson index ranged from 1.6 to 130.3 (Fig 4; S3 Table).

Fecal samples also showed high intra-individual variation in microbiota richness, represented by both the number of observed OTUs (ranged between 714 and 1 581; Fig 1B; S4 Table), and the Inverse Simpson index (21.5 to 167.1; Fig 4; S4 Table). The mean values of Inverse Simpson indices of raw milk are significantly lower than those of feces (mean = 86.4; df: 151.6, t-value: -5.575, P <0.0001).

### Factors affecting microbiota richness of raw milk and fecal samples

According to the results of the multi-model inference procedure (estimates of model-averaged parameters over the full set of models and their importance), the most important predictors explaining microbiota richness (alpha diversity expressed by Inverse Simpson index) in raw milk samples were percentage fat, following by diet and TBC (Table 1). Pearson’s correlation between observed and fitted values computed using model-averaged parameters was 0.35 (df = 61, P = 0.004) and the root mean squared error (RMSE) was 30.5. If the computation of the fitted values also included the random effect estimate for each herd, the correlation between observed and fitted values almost doubled (estimate = 0.67, df = 61, P < 0.001) while RMSE decreased to 23.8.

**Table 1:** Mean LMM estimates of multi-model inference parameters of raw milk microbiota composition.

Parameter	Mean estimate (95% confidence interval)	Importance
Intercept	58.90 (42.26–75.54)	-
Percent fat	5.79 (-6.05–17.63)	0.62
Diet (UF)	5.22 (-15.17–25.62)	0.36
TBC	1.14 (-4.25–6.54)	0.31
SCC	0.94 (-4.34–6.21)	0.29
Calving date	0.30 (-3.58–4.17)	0.24
Percent protein	0.01 (-4.52–4.54)	0.24

Parameters include intrinsic (i.e. calving date, total bacterial count (TBC), somatic cell count (SCC), percentage fat, percentage protein) and extrinsic (i.e. diet and herd) factors. UF: Unifeed diet or total mixed ration with silage.

Instead, fecal microbiota richness was explained by diet, on average UF diet was associated with greater diversity, followed by calving date and infection with coccidia (Table 2). Pearson’s correlation between observed and fitted values computed by model-averaged parameters was 0.33 (df = 97, P < 0.001) and the RMSE was 30.3. If the computation of the fitted values also included the random effect estimate for each herd, the correlation observed and fitted values greatly increased (estimate = 0.79, df = 97, P < 0.001) while RMSE decreased to 19.7. Model-averaged residuals of both LMMs did not show any violation of model assumptions.

**Table 2:** Mean Linear Mixed Model (LMM) estimates of multi-model inference parameters of fecal microbiota composition.

Parameter	Mean estimate (95% confidence interval)	Importance
Intercept	80.13 (58.98–101.29)	-
Diet (UF)	12.19 (-18.08–42.46)	0.53
Calving date	-1.41 (-5.78–2.96)	0.45
Coccidia	0.08 (-5.72–5.87)	0.25

Parameters include intrinsic (i.e. calving date, presence of Coccidia) and extrinsic (i.e. diet and herd) factors. UF: Unifeed diet or total mixed ration with silage.

The LM analysis of microbiota composition (also based on the Inverse Simpson Index) showed that explicitly considering ‘herd’ as a covariate (S5 Table and S6 Table) improved model performance for both milk (Pearson’s correlation = 0.68, df = 61, P <0.001 and RMSE = 23.8) and fecal (Pearson’s correlation = 0.79, df = 97, P <0.001; RMSE = 19.5) models, and confirms that intrinsic characteristics of each herd are highly correlated to the individual microbiota composition of the constituent cows.

### Factors affecting microbiota composition of raw milk and fecal samples

**Fig 5:**
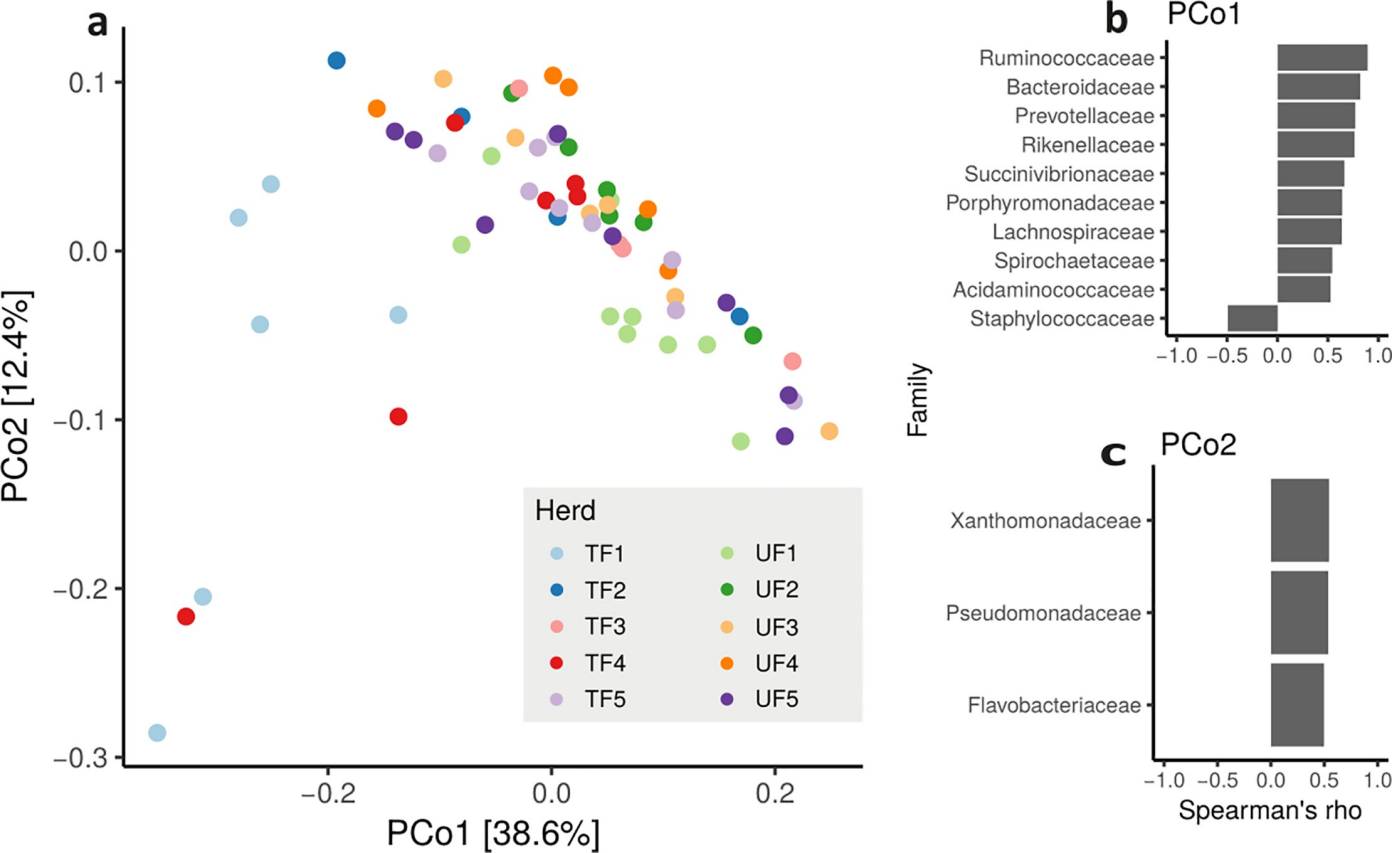
Estimates of raw milk microbiota composition of commercial Alpine dairy cows. (a) Principal Coordinate Analysis (PCoA) of the between samples distances measured using Bray-Curtis dissimilarity; Families that drive variability in raw milk microbiota composition in the first (b) and second (c) principal component. Colored dots identify cows from ten different herds either fed traditionally (TF) or with unifeed (UF) from the Province of Trento, Italy.

We inferred differences in microbiota composition of both raw milk and fecal samples with Bray-Curtis dissimilarity indices (beta diversity). The PCoA of raw milk microbiota composition did not show correlations between any factors investigated. However, when we applied the Permutational Multivariate Analysis of Variance (PERMANOVA), ‘herd’ explained the differences in milk microbiota composition between cows (P < 10^−4^; Fig 5A; S7 Table). The first principal coordinate (PC) of the PCoA (outlying cows on the left side of Fig 5A) is mainly driven by a greater abundance of *Staphilococcaceae* (Fig 5B), while the majority of the other cows (cluster in top right Fig 5A) have a greater abundance of nine other families, especially *Ruminococcaceae*, *Bacteroidaceae* and *Prevotellaceae* (Fig 5B). The second PC is mainly driven by differences in three bacterial families *Xanthomonadaceae*, *Pseudomonadaceae* and *Flavobacteriaceae* (Fig 5C).

**Fig 6:**
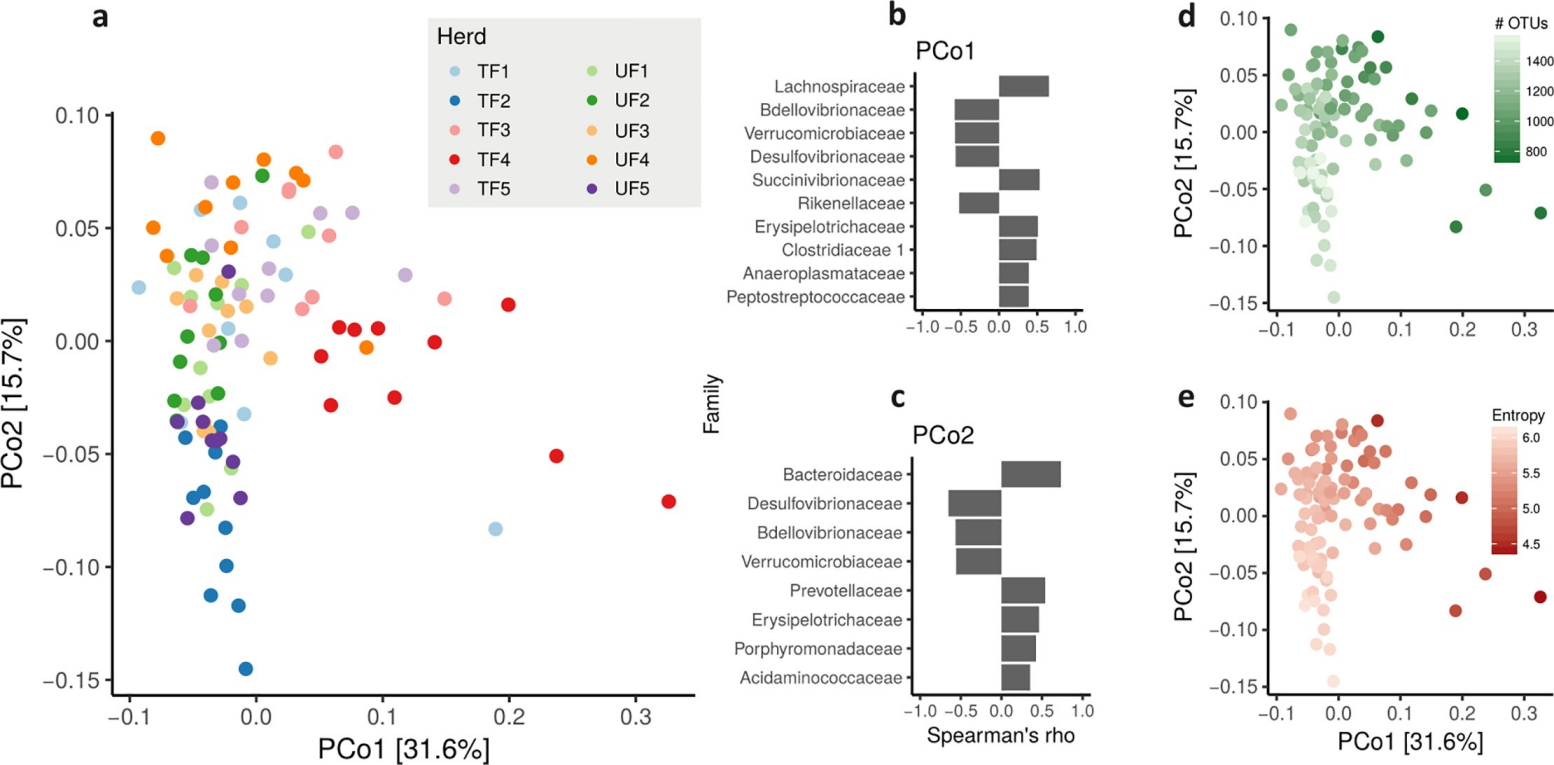
Measures of fecal microbiota composition of Alpine dairy cows. (a) Principal Coordinate Analysis (PCoA) of the between sample distances measured using Bray-Curtis dissimilarity; Families that drive variability in fecal microbiota composition in the first (b) and second (c) principal component; Sample distance based on the number of observed OTUs (d) and Shannon entropy (e). Colored dots identify cows from ten different herds either fed traditionally (TF) or with unifeed (UF) collected within the Province of Trento, Italy.

Similarly, for fecal samples, the PERMANOVA test showed that among all the parameters considered, only ‘herd’ was statistically significant (P <10^−4^; Fig 6A; S8 Table). The first PC (outlying cows on the right side of the Fig 6A, mainly from herds TF3, TF4 and TF5) is explained by a greater abundance of *Lachnospiraceae* and *Succinivibrionaceae*, whereas the majority of cows clustered on the left are characterized by a greater abundance of *Bdellovibrionaceae*, *Verrucomicrobiaceae*, *Desulfovibrionaceae* and *Rikenellaceae* (Fig 6B). Moreover, the feces of dairy cows at the top left of the graph (mainly from herds UF2, UF3, UF4 and TF5 in Fig 6A) are characterized by a greater number of *Bacteroidaceae* and *Prevotellaceae*, while those from herds TF2 and UF5 have more *Desulfovibrionaceae*, *Bdellovibrionaceae* and *Verrucomicrobiaceae*. Measures of bacterial richness showed that fecal samples with a greater number of OTUs (Fig 6D) also have a greater Shannon entropy (Fig 6E).

## Discussion

To our knowledge, this is the first large assessment of raw milk and fecal microbiota in commercial (non-experimental) dairy cows. We investigated almost 100 healthy cows from 10 small (non-intensive) Alpine farms, and explored how intrinsic and extrinsic factors may influence their microbiota richness and composition. Although variation between individual cows for both sample types was evident, we showed that ‘herd’ is the most important factor influencing individual differences in microbiota richness and composition in both raw milk and feces. Such knowledge could affect the use of microbiota manipulation for economic or veterinary purposes.

Despite belonging to different farms, breeds and feeding regimes, all raw milk and fecal samples of this study were dominated by the phylum Firmicutes, followed by Bacteroidetes and Proteobacteria. This result is in line with previously published studies of bovine raw milk [2, 4, 11, 48] and fecal samples [16, 18, 21, 24] in experimental herds or much smaller numbers of dairy cows. Furthermore, these three phyla are the most abundant in a variety of different mammalian species, such as human and non-human primates, laboratory mice [49–53], as well as wild herbivores (giant panda [54]; sika deer [55]; forest musk deer [56]). In addition, the low relative abundance of Proteobacteria found in the fecal samples of this study (2.5%) compared to Firmicutes and Bacterioides (89.6%), may reflect the health status of the cows. Healthy cows have reported to have less than 4% of Proteobacteria, while an increase of this relative abundance has been associated with subacute ruminal acidosis [24, 57, 58].

In our fecal samples from healthy cows, more than 50% of OTUs were Firmicutes while about 40% were Bacteroidetes (mean overall Firmicutes/Bacteroidetes (F/B) ratio = 1.34). Previous studies have shown that the F/B ratio in fecal samples often varies as a result of diet, in relation to digestion efficiency. For example, in humans, Firmicutes is primarily associated with energy harvest from food, while Bacteroidetes is linked to the production of short chain fatty acids, which provide cellular energy, maintain the epithelial barrier of the gut and modulate the immune system [52]. Elevated F/B ratios have also been linked to the development of obesity and metabolic disease in humans and genetically obese mice [59–62]. However, other studies were unable to confirm this correlation, and more recent reports concluded instead that such variation is influenced by several other factors [52, 63], and might be an evolutionary constraint of a particularly efficient gut [64]. Although the F/B ratio found in our healthy cows was within the range of previous investigations of commercial bovine herds [0.8 to 5.99; 18, 19, 23, 25], we did not find any correlation between the F/B ratio and other factors, such as diet (TF vs UF). Therefore, although the F/B ratio would be a simple and useful indicator of health, this correlation in domestic livestock has yet to be adequately investigated.

At the family level, *Ruminococcaceae* and *Lachnospiraceae* dominated both milk and fecal samples. Despite their important role in degradation of starch and fiber [15, 18], these two families are associated with gut health in various mammalian species, including horses [65], cats and dogs [66, 67] and laboratory mice [48, 68], as well as bovines [21]. Interestingly, despite variation between individuals and farms, we note for the first time that the same dominant families are also found in raw milk, which has important implications for calf and human gut health and nutrition. Other abundant families we reported in raw milk were *Staphylococcaceae*, commonly recovered from dairy cows [69], and *Corynebacteriaceae*, which has a wide range of fermentative capacities [70]. Among the most abundant families in feces, we found *Prevotellaceae*, which varies widely in response to dietary treatments [19, 71], and *Bacteroidaceae*, including some *Bacteroides* species that are known to break down cell wall components [72].

Among the abundant genera found in raw milk, we confirmed the presence of *Staphylococcus*, which is frequently found in other studies of bovine milk, as well as human milk from healthy individuals [11, 73, 74], despite the genus including well known pathogens like *S*. *aureus*, as well as many opportunistic disease agents. Although the roles of other milk and fecal genera are relatively unknown in bovines, *Bacteroides* and *Clostridium* XIVa have also been found in the intestinal tracts of most healthy mammals [72]; however, like *Corynebacterium*, their roles in milk have not been elucidated. Taxonomically heterogeneous *Clostridium* XI includes pathogenic species like the *C*. *difficile* which is commonly found on dairy farms. However, it does not apparently cause the increasing number of human cases of intestinal disease related to this pathogen [75]. Instead, *Acinetobacter* has previously been associated with mastitis [21, 76]. Notably, the composition of the core genera in raw milk varies widely between studies [4, 10, 11, 77] emphasizing that the role and function of specific taxa requires further investigation. Although we could identify a common core microbiota for all analyzed samples, we found high individual variation in terms of relative abundance of various taxa, even between animals belonging to the same herd. For example, some individual milk samples are clearly dominated by one genus (notably *Staphylococcus* or *Enterococcus*), whereas others displayed a more balanced profile (Fig 4A). Such intra-herd variation has already been reported by other authors [4, 23, 25].

In feces, the dominant genera included *Bacteroides*, *Alistipes*, *Prevotella* and *Paraprevotella* (*Bacteroidetes*); as well as *Clostridium* XIVa, *Roseburia* and *Phascolarctobacterium* (Firmicutes). In addition, *Treponema* (Spirochaetes) was also present. All these genera have been consistently reported for bovine feces in healthy individuals in previous studies. This present larger study confirms that, even though the relative abundance of the principal genera varies considerably among the different studies, these taxa could be considered useful biomarkers of healthy cows [18, 20, 24]. In particular, *Bacteroides*, *Clostridium* and *Roseburia* are known as ‘rumen digestion bacteria’, promoting digestion of complex organic matter and nutrient absorption [78], although various *Clostridium* are pathogenic or affect productivity [75, 79]. However, some *Clostridium XIVa* bacteria produce butyrate, which is responsible for eliciting an anti-inflammatory response, establishing and reducing intestinal permeability, and may even be involved in the prevention of colorectal cancer in humans [80–83]. *Bacteroides* spp. are also well-known mammalian intestinal bacteria [84, 85], and have been shown to form a resistance barrier against pathogens such as *C*. *difficile* by competing for monomeric sugars [78, 86], although other strains are pathogenic. *Roseburia* may contribute to producing butyrate that is used as the energy source for the intestinal mucosa [87]. While several studies on experimental [25, 18, 71] and commercial animals [19] have noted that the relative abundance of *Prevotella* and *Bacteroides* is frequently associated with dietary components, our study of commercial herds found no correlation between these genera and diet. However, in the current study, diets were only different in forage type and processing (hay or silage), not nutritional components.

Interestingly, in fecal samples we observed that samples characterized by a greater number of OTUs were more similar to each other (tend to cluster together) and have a higher entropy, whereas samples characterized by fewer OTUs were the most variable, with a lower entropy. Several authors have shown that more stable and productive communities, including human gut microbiota [88], but also zooplankton [89, 90] have better pathogen resistance. Other authors have noted that decreases in microbiota richness often accompany disease, and lower richness may lead to a more limited ability of the microbiota to respond to different stressors [91, 92]. However, lower richness is not always correlated with lower fitness in non-human animals [93, 94], and in fact, we did not find an association with the presence of coccidia and Inverse Simpson index here. Therefore, the ‘protective’ capacity of a highly diverse microbiota could depend on the type of pathogen. Further work is needed to elucidate the role of microbiota diversity and protection against parasites.

Although there are several studies that attempt to identify the factors that influence the milk and fecal microbiota in cattle, the majority of these focus on animals from single experimental farms, and a single or a few factors at a time. Instead, this study modelled simultaneously which extrinsic and intrinsic parameters correlated with the composition of milk and fecal microbiota in dairy cows from 10 commercial farms. Despite choosing farms with similar management, our results showed that every herd had its own characteristic microbial community, and overall, the farm-to-farm differences seen here confirm previous but much smaller studies of dairy herds for feces and suggest that local farm management is unique and can significantly influence diversity and composition even at microbiological level [23, 26]. In fact, the microbiota of each farm could be influenced by a number of management choices such as sources of dietary ingredients, water supply, bedding material, hygiene practices, use of outdoor environment (e.g. pastures and soil types), and milking hygiene. Whereas the impact of individual variants on fecal and milk microbiota are frequently studied, the influence of management types needs further study. Since the understanding of functional microbiota in non-human animals is in its infancy [93] we can also say very little at this time about the significance of variations in specific microbiota components. For example, although some *Staphilococcaceae* may be pathogenic, *Bdellovibrionaceae* could have an importance in the defense against animal pathogens [95], and *Desulfovibrionaceae* produce cellular energy [96], much work remains to be done to understand the roles of various microbiota compositions and whether they have an effect on livestock production and health. Interestingly, the ‘herd factor’ has recently been shown to be important in the immune and inflammatory responses in dairy cows [97, 98, 99], thus it would be highly interesting to test whether the microbiota is involved in these responses, given that it plays a fundamental role in development of the host immune system [100]. More importantly for veterinary medicine, however, these results imply that the effect of antibiotics or probiotics on microbiota may also vary between farms; that is, both testing and applying new treatments should take differences in herd microbiota composition into account, since efficacy could been linked to farm management rather than the effect of the therapy.

Other than herd, important predictors of Inverse Simpson indices were percentage fat for raw milk, and diet for both raw milk and fecal samples. Interestingly, milk microbiota richness is associated strongly with milk fat content. Since modern breeds have been selected for higher milk quantity, this has led to milk with lower fat content. Low fat milk is also considered a ‘healthier’ choice by today’s consumers. Our result suggests the importance of fat in milk microbiota richness; thus if such diversity is considered beneficial to health, then lowering fat could have an effect on both animal health and human nutrition. Instead, the link between richness and diet is not surprising, since many authors consider diet, in terms of diet formulation, feeding management and fecal starch concentration as the greatest factors altering fecal bacterial communities [13, 18, 19, 71].

## Supporting information

S1 Table Taxonomic profile at phylum level for raw milk samples. (PDF)

S2 Table Taxonomic profile at phylum level for fecal samples. (XLSX)

S3 Table Metadata of observed OTUs and Inverse Simpson index associated with raw milk samples used in this study. (XLS)

S4 Table Metadata of observed OTUs and Inverse Simpson index associated with fecal samples used in this study. (XLS)

S5 Table Results of Linear Model (LM) examining the influence of parameters on milk microbiota composition with ‘herd’ considered as a covariate. (DOCX)

S6 Table Results of Linear Model (LM) examining the influence of parameters on fecal microbiota composition with ‘herd’ considered as a covariate. (DOCX)

S7 Table Permanova test: Milk. (XLSX)

S8 Table Permanova test: Feces. (PDF)
