# Supplementary material for: Raw milk and fecal microbiota of commercial Alpine dairy cows varies with herd, fat content and diet
Source: PLoS One. 2020 Aug 6;15(8):e0237262. doi: 10.1371/journal.pone.0237262 (PMC7410245; doi:10.1371/journal.pone.0237262)
Supplement: S5 Table — (DOCX) [file pone.0237262.s005.docx]

| **Parameter** | **Importance** |
| --- | --- |
| Herd | 0.98 |
| Percentage fat | 0.58 |
| Percentage protein | 0.19 |
| Calving date | 0.18 |
| TBC | 0.18 |
| SCC | 0.17 |
