# Supplementary material for: Raw milk and fecal microbiota of commercial Alpine dairy cows varies with herd, fat content and diet
Source: PLoS One. 2020 Aug 6;15(8):e0237262. doi: 10.1371/journal.pone.0237262 (PMC7410245; doi:10.1371/journal.pone.0237262)
Supplement: S8 Table — (PDF) [file pone.0237262.s008.pdf]

Faeces

Call:

adonis(formula = d ~ heard.ID, data = v, permutations = 9999)

Permutation: free

Number of permutations: 9999

Terms added sequentially (first to last)

	Df	SumsOfSqs	MeanSqs	F.Model	R2	Pr(>F)
heard.ID	9	0.63508	0.070564	6.9418	0.41245	1e-04 ***
Residuals	89	0.90469	0.010165		0.58755	
Total	98	1.53976			1.00000	
